# One Year of the COVID‐19 Pandemic. What Do We Know and What Is Yet to Come? — The Summarising Review

**DOI:** 10.3389/ijph.2021.1603975

**Published:** 2021-07-30

**Authors:** Wojciech Malchrzak, Agnieszka Mastalerz-Migas, Zbigniew Sroka, Maciej Spiegel

**Affiliations:** ^1^ Department of Family Medicine, Wroclaw Medical University, Wroclaw, Poland; ^2^ Department of Pharmacognosy and Herbal Medicines, Wroclaw Medical University, Wroclaw, Poland

**Keywords:** public health, COVID-19, coronavirus, pandemic, SARS-CoV-2, 2019-nCoV, pneumonia, epidemic

## Abstract

**Objectives:** The aim of this review is to summarize the most relevant scientific discoveries regarding SARS- CoV-2 virus infection, with the special emphasis put on its pathophysiology and way of treatment.

**Methods:** In November 2020, the research articles have been collected and examined manually to pick the most relevant. In case of fresh topics, e.g. vaccines, we have performed searching using adequate keywords. Preliminary analysis was conducted on 200 manuscripts.

**Results:** Among them 59 papers were out-of-scope, and thus were rejected from the further elaboration. Another 25 papers were rebuffed because they presented topics, that have been extensively described in the already included papers. Basing on the 29 papers we have estimated ratio of observed SARS-CoV-2 infection clinical manifestations and comorbidities among hospitalized patients. 12 papers let us evaluate frequencies of deviations within laboratory markers concentrations, as well as weighted average of the laboratory tests results.

**Conclusion:** Due to the significant infectivity of the virus and its harmfulness towards organism further studies are required to find accurate way of the disease treatment and suspending its spreading.

## Introduction

The coronavirus disease (*COVID-19*) is caused by the Severe Acute Respiratory Syndrome Coronavirus 2 (*SARS-CoV-2*) which belongs to the *Coronaviridae* family. The newly-mutated virus has proven to be more lethal than any other coronavirus before it and as of November 22, 2020, nearly 57.8 million people had become infected by SARS-CoV-2 and approximately 1.4 million of them had died since the start of the pandemic [1]. In this narrative review, we summarise the most important and most frequently cited papers on the coronavirus released within the first year of the pandemic to establish what has been done so far and what are the outcomes, as well as to refer to novel approaches that make further studies easier and let the world eradicate the ‘Black Death’ of the 21st century. This is most likely the very first paper addressed to medical professionalists, and as such, it provides information useful for clinicians, general practitioners, pharmacists, and other medical professions engaged in unfair competition with the virus.

## METHODS

### Search Strategy

The literature search was performed through Web of Science database in order to find relevant original articles or case series concerning COVID-19 disease. In order to focus on the most important papers the additional filter, “Hot paper”, was applied in the search engine [2]. The articles have been gathered in November 2020 and the results consisted majorly of the papers published online between January and August 2020. No additional restrictions narrowing area of search were put into the search engine. Finally, the results were sorted by the number of citations, starting from the highest cited one.

### Inclusion and Exclusion Criteria

First 200 papers from the results list were briefly examined at the beginning. Reviews, systematic reviews, meta-analyses, recommendations and pure mathematical models were excluded. If the article presented worthful discovery about SARS-CoV-2 pandemic, the database was additionally searched by the original article keywords to check whether any other study may expand the topic. This approach has been found to be especially relevant when treatment plans or spread prevention methods were under consideration. This arises from the dynamic changes in drug administration policies and continuing studies on COVID-19 eradication. The data regarding morbidity and mortality were taken from the most recent WHO report at the day of this paper finalization.

### Article Selection and Data Extraction

The abstracts and full texts of the first 200 research papers were screened independently by W.M. and M.S. —articles were in-depth analysed to evaluate their usefulness in the subject of the review. Final choice was made after discussion and consensus. This led to rebuff of 59 papers (29.5%). At this stage additional 25 papers (17.73% of the remaining) were rejected, because their scope was already fulfilled by other, already included manuscript. We have focused on papers that presented facts important for medical professionals, also these including clinical data of the patients—they were used to calculate frequencies of certain comorbidities and clinical manifestations (29 papers) as well as laboratory markers deviation (12 papers). In the end 40 articles were rejected due to their detailed aspects that cannot be summarised without a significant lose to their value.

### Data Analysis

The collected data was split and grouped to the subjects presented in this review—pathophysiology, infectivity and detectability, clinical manifestation, diagnostic and medical treatment. Qualitative data were synthetically presented preserving consistency of pathological mechanisms and aberrations resulting from them. Quantitative data of existing comorbidities and clinical manifestations were processed to obtain averaged values for samples size. For the laboratory markers data, a weighted arithmetic means of median values presented in the papers were calculated.

## Results

We summarized the results of one year of scientific study on SARS-CoV-2 in this work by reviewing the provided number of research papers. The literature review enabled us to identify the most important characteristics of SARS-CoV-2 infectivity and the COVID-19 illness itself. With such informations, it was feasible to write a manuscript that focused on the practical element — specifically, the role of COVID-19 pathogenesis — since understanding the progression of the disease can help with diagnosis and therapy. This was complemented by clinically relevant data, such as the frequency of clinical symptoms, the prevalence of comorbidities among hospitalized patients, and the usual levels of laboratory markers among infected people. Furthermore, fields that have yet to be found have been highlighted in the text. Everything in a clear, devoted to medical professions format.

## Discussion

### Pathophysiology

The key symptoms seen during the first days of a SARS-CoV-2 infection are generally related to the respiratory system, which suggests its extended tissue tropism [3]. Animal studies confirmed that the virus is excreted from type I and II pneumocytes as well as ciliated respiratory system epithelial cells [4]. Using the transgenic-mice model with expressed human angiotensin-converting enzyme 2 (*ACE2*) receptors, Bao et al. [5] observed high affinity of the virus for them. Their number varies depending on the tissues—the predominant sites are the small intestine, heart and kidneys whilst lungs demonstrated a comparably lower ACE2 expression profile [6–9]. However, this does not explain such things as anosmia, which is a typical COVID-19 symptom. Aside from the fact that the respiratory tract is the most accessible transmission approach, recent studies suggest that ACE2 and transmembrane serine protease 2 (*TMPRSS2*) must work together in the viral uncoating mechanism—they are significantly co-expressed e.g., in the nasal cavity or type II pneumocytes [7, 8, 10]. To adhere to the host’s cell, the virus uses its receptor-binding domain allocated in the S1 subunit of the trimeric spike (S) glycoprotein, expressed on the surface of the viral envelope. Upon binding with ACE2, TMPRSS2 primes the S1-S2 junction, presenting another subunit (S2) responsible for the membranes’ fusion [3, 11].

The uncontrolled viral infection leads to macrophage and lymphocyte infiltration and accumulation in the alveolar interstitium [5, 12]. The endothelium of the damaged lungs releases procoagulant factors, e.g., von Willebrand factor and Factor VIII, and pathological fibrin clot formation takes places in the alveolar spaces. This process is further amplified due to the impairment of the fibrinolytic functions caused by plasminogen activator inhibitor-1 upregulation deriving from the interference between the NSP1 and ORF6 viral proteins and the signal transducer and activator of transcription (*STAT1-STAT3*) cascade [13, 14]. Fibrin clots formed in alveolar capillaries by the complement system were found to cause injuries of the interalveolar septum, and consequently, diffuse alveolar damages [14, 15]. Due to the hypoxic vasoconstriction (or even complete blockage of blood vessels) and activation of hypoxia-inducible factors, blood flow becomes drastically reduced, which explains the breathing problems that are common among the hospitalised patients, as well as hypoxia being a major cause of death [14, 16, 17].

The viral sepsis mechanism is multifactorial and depends on the cytokine storm, microcirculation dysfunctions and virus dissemination. Activated immune cells excrete cytokines and inflammation mediators, mainly IL-6; the increase of its levels in the plasma results in the cytokine storm syndrome. Upon reaching other organs through the haematogenous route, cytokines induce local inflammation. Such condition—if not remedied quickly enough—may evolve into a more critical systemic inflammatory response [12, 14, 17, 18]. It is worth noting that increased interferon type I (*IFN-1*) concentration observed during the infections seems to upregulate the ACE2 gene expression and enable easier transmission of the virus between host cells [8, 19]. Through the damage in bronchi, the virus gains access to vascular endothelial cells and then to the bloodstream where it resides [20].

A reverse sequence takes place when other organs are infected. For example, renal tissue damage is claimed to be an outcome of three different ways of action: 1) the previously–mentioned ACE2–TMPRSS2–dependant pathway and direct cytopathic effect; 2) by interaction with immune cells and the immunological effect mechanism (immune complex, cytotoxic effect); 3) indirectly by the pathological activity of the released cytokines [20, 21]. Furthermore, hypercoagulability is inseparably associated with blood clot formation manifested by lower limb deep vein thrombosis and pulmonary embolism [22, 23]. This happens even while therapeutic anticoagulant doses are administered [14].

One of the most specific and clinically relevant SARS-CoV-2 infection symptom is sudden anosmia and hyposmia (56.97% of infected patients [24]). Their development might be linked to the viral invasion of the nasal cavity, which disrupts the functioning of the olfactory epithelium and olfactory nerve. The olfactory epithelium and olfactory nerve also seem to be the starting points of the central nervous system (*CNS*) infection, which are different from the hematogenous route—the virus might enter the CNS through the retrograde neuron pathway via olfactory bulb [3, 24, 25]. However, further studies are required to explain this problem.

Since the virus exhibits immunosuppressive activity, it weakens hosts’ natural defence system [26]. The antibody–dependant enhancement (*ADE*) mechanism was observed in patients who had previously been exposed to non-SARS-CoV-2 coronaviruses. It relies on a quick but inappropriate immune response which leads to an increase of viral load. It may results in a sustained cytokine storm and inflammation which are manifested in the clinical course among the patients [27, 28]. For these reasons, we suppose that ADE might be involved in the more severe course of the reinfections. Concluding, all mechanisms presented may together contribute to the severity and lethality of the disease course.

### Infectivity and Detectability

To et al. [29] found that the viral load correlated positively with age and Zheng [30] determined that virus duration was longer in older male patients. The basic reproduction number—expressing the expected number of new cases generated by a single case—estimated for the Diamond Princess cruise ship passengers by Zhang [31] is 2.28. In our opinion, the statistics presented are reliable because samples were taken from a large and closed group (3,711 samples), which was not affected by other factors.

The results presented by Wölfel [3] indicate that viral load was mostly found in the sputum, making it an efficient aerosol or droplets transmission carrier, e.g. though coughing or sneezing. Recently, airborne and fomite–based transmissions also suggested as well [3, 32]. These reports indicate that special attention should be paid to tracheal intubation as it increases the risk of the exposure to viral loads suspended in the air, and consequently, superinfection incidents. Aside from the respiratory tract, samples of viral mRNA were also found in the serum (16 days since the first detection [30]) and stool (22 days [30]; 11 days [33]; average = 16.5 days), indicating the virus may replicate in the gastrointestinal (*GI*) tract.

### Clinical Manifestation

We divided the disease course into three general phases based on clinical manifestation — 1) asymptomatic or presymptomatic; 2) symptomatic; 3) post-symptomatic.

Phase 1) begins when the virus enters the host and starts to replicate. This phase finishes upon the first symptoms or when the virus vanishes in the case of the asymptomatic patients. The virus incubation period, estimated based on a group of 262 patients located in Beijing, was determined to be about 1 week [34]. Nonetheless, the virus can still be transmitted even if reaching the peak viral load is delayed. He et al. found that over half of the infections occurred during the presymptomatic phase [35]. Since the Diamond Princess study tells us about 17.9% of the asymptomatic patients [36], isolating persons who potentially had contact with patients who have tested positive is justified.

Phase 2) starts after the first symptoms and lasts throughout the respiratory tract symptoms and/or fever.

**FIGURE 1:**
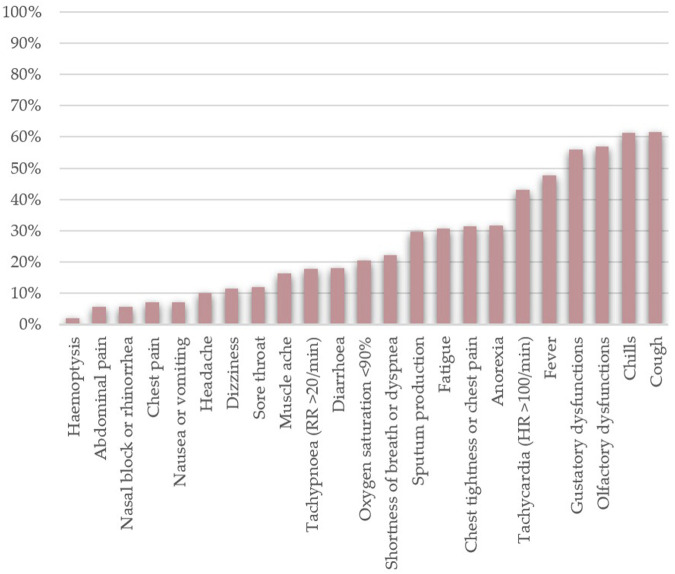
Frequencies of the most common clinical manifestations of SARS-CoV-2 infection found among hospitalised patients (n = 17,444). The data was collected from the following papers — [3, 15–17, 21, 24, 26, 29, 30, 34, 37, 38, 41–43, 45–47, 49, 52, 55, 67–69, 73–75, 77, 79].

Phase 3) occurs directly after phase 2) when acute symptoms subside. Patients in phase 3) can still manifest symptoms—not directly linked to viral replication—and excrete enough viral load to infect others [3, 29, 36]. Figure 1 shows the common SARS–CoV–2 infection symptoms that patients complained about, along with their frequency.

An interesting observation regarding the virus activity can be made based on the studies presented [17, 37, 38]. Among the patients included in the trials, second–generation hosts were less likely to die. This would suggest that the longer the virus is present in the population, the further its mortality lowers. It may be connected with the virus evolution process—with the loss of pathogenicity, it increases its transmissibility, and consequently, the number of infected hosts.

No differences were found between pregnant and non–pregnant patients; neither foetal malformations nor birth injuries were observed [39]. The disease course in children is likely to be similar to asymptomatic patients or be accompanied by cold/flu symptoms—fever, cough, shortness of breath [40]. While occasionally present, complications are rather uncommon.

As for now, it has been observed that post–symptomatic patients suffer from long–term complications due to SARS-CoV-2 derived lungs fibrosis, i.e., persistent fatigue and decreased respiratory capacity. Further observations are required to recognise other ones.

#### Increased Morbidity Risk Factors

**FIGURE 2:**
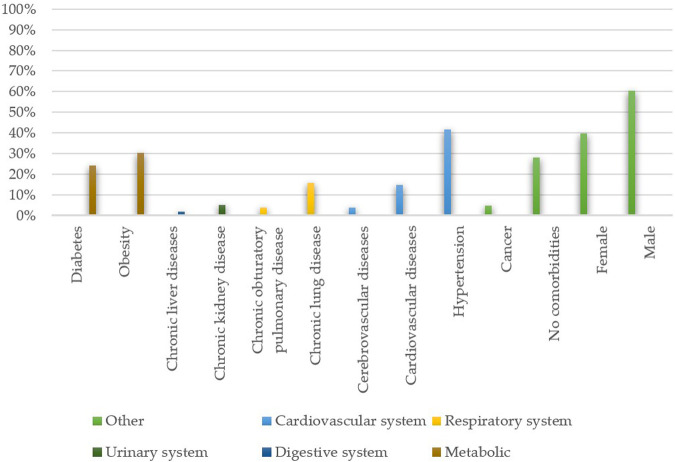
Frequencies of the existing comorbidities and patients’ sex among hospitalised patients (n = 17,444). The data was collected from the following papers — [3, 15–17, 21, 24, 26, 29, 30, 34, 37, 38, 41–43, 45–47, 49, 52, 55, 67–69, 73–75, 77, 79].

Based on the papers, both older age and being male (as shown in Figure 2) can be deemed risk factors [41–43]. The existence of comorbidities is generally known to weaken the host’s defence system and increase infection severity, which may lead to a poorer clinical outcome. A similar correlation exists in the case of COVID-19 and is particularly evident. The coexistence of comorbidities (Figure 2) has a multifunctional impact on the prognosis, for example:

1) Increased body mass index has been linked to the higher ratio of invasive mechanical ventilation and intensive care requirements [44];
2) Patients with diabetes found themselves in a virtuous cycle as the disruption of the blood glucose levels due to viral infection impedes the recovery process, probably due to the damage in the pancreas [45];
3) Elevated baseline serum creatinine is a risk factor for hospital admission, mechanical ventilation and acute kidney injury (*AKI*) development. AKI manifested itself as a shock, rhabdomyolyses and hypoxia [21];
4) Among patients with the GI tract dysfunctions an elevated sputum production accompanied by increased levels of lactate dehydrogenase and glucose indicated a worse clinical course of the disease [46];

Based on the WHO data [1], we estimated that the global mortality rate is approximately 2.88% and 3.57% for Europe.

### Diagnostic

Table 1 shows the list of all diagnostic markers. A sufficiently quick diagnosis is vital for both the patient prognosis and eradicating the epidemic. Many valuable diagnostic tools can be used for this purpose. For example, in several studies, an increased D-dimer concentration (>1.0–2.0 μg/ml) —a blood clot degradation product—was an independent predictor of death due to the SARS-CoV-2 infection [47–49].

**TABLE 1:** Laboratory findings among COVID-19 patients. The data was collected from the following papers — [17, 21, 29, 38, 43, 47, 49, 67–69, 75, 77].

Marker	Median value	Marker deviation	% Of patients
White blood cells	7.68 × 10^9^/L	>9.5 × 10^9^/L	15.86
Neutrophiles	5.19 × 10^9^/L	>5.8 × 10^9^/L	24.74
Lymphocytes	0.99 × 10^9^/L	<1.5 × 10^9^/L	58.32
C-reactive protein	13.46 mg/dl	↑	75.55
Procalcitonin	0.25 ng/ml	↑	27.89
Erythrocyte sedimentation rate	32.50 mm/h	↑	81.60
Asparagate transaminase	49.44 U/L	>40 U/L	50.47
Alanine transaminase	36.26 U/L	>40 U/L	34.37
Albumin	32.89 g/L	<32 g/L	45.40
Creatinin	111.64 μmol/L	↑	8.05
D-dimer	596.73 ng/ml	↑, >0.5 ng/ml	61.45
K^+^	12.21 mmol/L	<3.5 mmol/L	23.50
Na^+^	152.39 mmol/L	>145 mmol/L	8.39
Platelets count	302.65 × 10^9^/L	<145 × 10^9^/L	12.09
Troponin	6.78 pg/ml	↑	23.37
NT-pro-BNP	375.20 pg/ml	>285 pg/ml	49.13
Lactate dehydrogenase	451.42 U/L	>245 U/L	61.54
Creatine kinase	175.96 U/L	>100 U/L	16.27
Prothrombin times	12.57s	>14.5s	31.81
APTT	31.14s	>42s	40.00
Ferritin	788.54 ug/L	>300 ug/L	79.69
Glucose	6.43	↑	78.82

Similarly, white cells profiles should be monitored systematically. Severe lymphopenia is also linked to the higher mortality—patients with total T cell, CD8^+^ T cells and CD4^+^ T cell counts lower than 800/μL, 300/μL and 400/μL, respectively, were more likely to die. Additionally, such CD4^+^ and CD8^+^ cells often expressed programmed cell death protein 1, which indicated their fatigue [50].

Long et al. [51] estimated that 17–19 days elapsed until the positive virus–specific IgG had reached 100%, whereas 20–22 days are required to reach a positive virus-specific IgM at the rate of 94%. The authors distinguished three seroconversion types: synchronous seroconversion of IgG and IgM; IgM seroconversion before IgG; and IgM seroconversion after IgG. Based on the real-time polymerase chain reaction from a sputum, the viral load slowly decreases after seroconversion occurs. Nonetheless, “slowly” is the keyword here because viral RNA is still detectable at least one week from the symptom onset and sometimes even longer [3, 29, 49], though the median time to the first negative test from the respiratory tract was 9.5 days [33]. The overall seroconversion rate was 96.8% in a group of 63 patients [51].

The radiological evolution of COVID-19 pneumoniae is a suitable tool for tracking the disease’s progress. Computed tomography (*CT*) makes it possible to observe respiratory tract injuries, which are particularly relevant in the diagnosis of asymptomatic patients with negative serum–antibodies assay. An attention should be paid to ground–glass opacities and consolidations [52]. CT scans might also make it easier to diagnose paediatric patients. The typical findings among youngsters are unilateral or bilateral subpleural ground–glass opacities and consolidations with reverse echo [40, 53]. Yet, this procedure is not advocated in the case of paediatric patients due to the possible harmful effects of X–ray radiation.

### Medical Treatment

#### Potentially Relevant Drugs

**Anticoagulants.** Patients undergoing sepsis–induced coagulopathy (SIC) criteria with more than four points should take anticoagulant drugs—unfractionated and low–molecular–weight heparins [48]. Pulmonary embolism, common morbidity developed during COVID-19, increases the risk of acute respiratory distress syndrome (*ARDS*) despite anticoagulant administration. Imbalance in the coagulation system is so significant that even therapeutical doses of the drugs are insufficient to restore the equilibrium [14, 54]. Preliminary researches on the tissue plasminogen activator indicates that fibrinolytic agents might be useful in severe COVID–19 cases [55].

**ACE inhibitors and angiotensin II receptor blockers*.*** Due to the participation of the ACE2 receptor in COVID–19 pathophysiology, it might be useful to utilise ACE inhibitors (*ACEi*) and angiotensin II receptor blockers (*ARB*s), which was confirmed by studies linking their administration to disease mitigation and a lower risk of all–cause mortality, especially if the patient had coexisting hypertension. ACEi/ARB lower the IL–6 concentration, which is responsible for the cytokine storm, and increase the number of CD3 and CD8 T cells in the blood, thus stabilising the immune system and decreasing the viral load [43, 56]. Yet, their preventive intake does not seem to lower the risk of infection [57, 58].

**Tocilizumab and anakinra.** IL-6 hyperactivity inhibition is another potential route towards developing a treatment. Tocilizumab administration has been linked to a better clinical course, overall condition stabilisation of the laboratory markers and improved CT scans among patients with cytokine storms caused by COVID-19. Moreover, no severe adverse effects were observed. This makes tocilizumab an interesting candidate to become a drug routinely used in SARS-CoV-19 treatment [16, 19, 59, 60]. Because of its similar mechanism of action, anakinra is also likely to be used [19].

**Convalescents plasma.** Based on the successive use alloantibodies in the treatment of chickenpox, hepatitis B and tetanus, there are high hopes for the convalescent plasma (*CP*) treatment. Being rich in virus-specific IgG and IgM (titres ∼1:640), the premise of such a therapy is a direct neutralisation of the virus through antibody activity. Clinical trials have demonstrated an improvement in breathing parameters and normalisation of the diagnostic markers. The positive effect of convalescent plasma was also shown in intensive care units. It was noticed that the patients to which it was applied had reduced hospitalization time, a lower risk of mechanical ventilation necessity, and vasopressive medication use was reduced or entirely stopped [61]. However, CP should undergo preliminary tests before the application due to the possible presence of sub-neutralising concentrations of immunoglobulins, and consequently, the potential appearance of the ADE mechanism [62, 63].

**EK1C4 (experimental).** Xia [64] studied the activity of the EK1 lipopeptide against SARS- and MERS-CoVs on the mice model to find ways of protecting people from coronaviruses. Among the compounds tested, EK1C4 was found to be the most potent against SARS-CoV-2. The drug binds to the S2 subunit of the coronavirus spike protein, effectively inhibiting membrane fusion and RNA release.

#### Supportive Therapy

**Glucocorticosteroids.** A meta–analysis conducted by Ye [65] shows that glucocorticoid drugs seem to decrease the mortality of patients with ARDS. However, in the early phase of the infection—when the viral replication and not inflammation is responsible for the clinical manifestation—glucocorticosteroids (*GKS*) intake might not be beneficial [66]. Interestingly, Chen et al. observed that patients with an ongoing cytokine storm who were administered GKS had a higher fatality ratio [17]. This may be due to the delayed GKS administration, which may have prevented it from stopping the already expanded inflammation.

**Intensive care unit treatment and oxygen therapy.** Respiratory impairment was the most common reason for intensive care unit admissions [42]. Patients commonly required intubation and mechanical ventilation carried out the high positive end-expiratory pressure [42]. Patients undergoing mechanical ventilation often had hypotension incidents and vasopressors had to be administered [67]. Approximately 1/4 of the patients also required oxygen therapy [68].

#### Ineffective Drugs

**Antibiotics.** Several studies tested whether antibiotics might combat the disease but they failed to help [46, 69]. While antibiotics show no direct antiviral activity, macrolides might be useful as they are claimed to modulate the immune system without having a direct influence on virus [70]. Azithromycin and its derivates are likely to eradicate bacteria responsible for further superinfections, but the treatment did not result in an improvement of the patients’ disease courses [71]. A similar situation took place during the MERS epidemic, when azithromycin administration did not lower the mortality rate [72]. For these reasons we see no purpose in administering antibiotics if there are no evident cases of secondary bacterial infection. Despite their preventive use, there is no evidence of their beneficial activity [17]. Finally, the increasing antibiotic resistance of bacteria and the side effects of antibiotics e.g., intestinal microbiota dysbiosis, are yet additional reasons to reconsider administering antibiotics.

**Chloroquine and hydroxychloroquine.** There is no clinically relevant evidence that either chloroquine or hydroxychloroquine should be administered when treating COVID-19, even though such rumours have spread recently. Instead of helping, both patients and medical personnel had to deal with newly discovered adverse effects of their use [41, 73, 74].

**Lopinavir/ritonavir.** A prospective randomised trial performed by Cao [75], as well as a retrospective study made by Zhou [49], did not find any beneficial effects when lopinavir/ritonavir was administered.

**Remdesivir.** A WHO SOLIDARITY trial [76] (*n* = 11.226) did not provide any indisputable proof confirming remdesivir’s activity—the confidence interval obtained indicates either prevention of some deaths or no such a dependence. For this reason, no recommendations on remdesivir’s usage were formulated by the WHO. Wang [77] conducted a clinical study (*n* = 237) where the disease course was not mitigated when compared with the placebo groups. Patients often manifested severe adverse side effects that required the withdrawal of the treatment plan. On the other hand, Beigel [78] (*n* = 1,062) noticed a decrease in mortality and median recovery time when the drug was administered. Based on these three papers, we conclude that using remdesivir may bring favourable results for some patients, and that is why studies on larger groups show much more promising outcomes. The necessity of conducting further studies on remdesivir—which is still being used by clinicians despite official guidelines—should be emphasized.

#### Vaccine

The main target of a vaccine is the S–protein, especially the S2 subunit, which is demonstrated to be the most important factor in the membrane fusion process. As for now, the most desirable immune response is the one deriving from the T lymphocytes activity. There are already antibodies and lymphocytes CD4^+^ that are directed towards this protein [79]. A report concerning the first monoclonal antibody capable of neutralising the virus using a mechanism independent of the receptor-binding inhibition was published recently. It has been claimed that not only does it prevent from the disease, but it also cures it [80]. At the moment, the EMA has conditionally approved four vaccines: two of them use viral mRNA embedded in a lipid shell, and two are adenovirus vectors devoid of replication capabilities. Each of them has a mechanism founded on S-protein production within host cells against which an immune response is being produced [81–84]. It was found to stimulate a response from T-helper cells and induce both cellular immunity and antibody production [85]. Despite of the starting vaccination program we still do not know what is ahead of us.

### Summary

Despite the fact that it has only been one year since the outbreak, the scientific goal of eradicating the virus has resulted in hundreds of publications of all types. As a result, the biggest constraint we are aware of is the difficulty of covering them all in this paper. Even as these lines are being written, a novel and scientifically important contribution may be reviewed. Nonetheless, owing to pharmaceutical companies' engagement in vaccine research, the prospects are promising, and we expect many amazing breakthroughs in the future.
